# Relationship between High School Mathematics Grade and Number of Attempts Required to Pass the Medication Calculation Test in Nurse Education: An Explorative Study

**DOI:** 10.3390/healthcare3020351

**Published:** 2015-05-27

**Authors:** Johanne Alteren, Lisbeth Nerdal

**Affiliations:** Faculty of Professional Studies, University of Nordland, Campus Helgeland, Postboks 614, N-8607 Mo i Rana, Norway; E-Mail: Lisbeth.Nerdal@uin.no

**Keywords:** nurse education, nursing students, medication calculation test, mathematical skills, attempts to pass the medication calculation test

## Abstract

In Norwegian nurse education, students are required to achieve a perfect score in a medication calculation test before undertaking their first practice period during the second semester. Passing the test is a challenge, and students often require several attempts. Adverse events in medication administration can be related to poor mathematical skills. The purpose of this study was to explore the relationship between high school mathematics grade and the number of attempts required to pass the medication calculation test in nurse education. The study used an exploratory design. The participants were 90 students enrolled in a bachelor’s nursing program. They completed a self-report questionnaire, and statistical analysis was performed. The results provided no basis for the conclusion that a statistical relationship existed between high school mathematics grade and number of attempts required to pass the medication calculation test. Regardless of their grades in mathematics, 43% of the students passed the medication calculation test on the first attempt. All of the students who had achieved grade 5 had passed by the third attempt. High grades in mathematics were not crucial to passing the medication calculation test. Nonetheless, the grade may be important in ensuring a pass within fewer attempts.

## 1. Introduction

Handling drugs in an appropriate and secure manner is important in ensuring patients’ health. Handling drugs is therefore an essential skill for nurses. In the medication administration chain, there is a risk of making mistakes, and medication errors are common in healthcare [1]. They are costly and detrimental to patients’ health [2]. Examples of these errors are incorrect drug or dosage administration; use of incorrect administration methods; administration of drugs at the incorrect time or to the wrong patient; prescription errors; and the occurrence of unexpected effects [3]. Each year, specialist health services in Norway report adverse events in medication administration to Meldeordningen, the Norwegian reporting system for adverse events in specialist health services [3]. Messages regarding events related to medication errors accounted for 19% of all reports. In addition, 2% of the medication errors reported involved unnatural deaths.

To reduce clinical errors, nursing education in Norway has increased its focus on improving knowledge regarding the subject. Nursing students are required to achieve a perfect score in a medication calculation test and handle drugs while practicing in the clinic. In accordance with the examination regulations at the university college, the medication calculation test is classed as an examination, which means that students are allowed four attempts to pass. During the exam, the students are permitted to use calculators but cannot use dosage calculation formulae. Another requirement is that students pass the test before undertaking their first practice period during the second semester. To facilitate this, the university college allows for four attempts in the examination schedule, which are to be made within a relatively short period. Prior to making the fourth attempt, students are required to apply for the exam and undergo obligatory supervision. The requirements are embodied in the national curriculum [4].

Several authors have described students’ levels of basic mathematics as a presenting challenge in passing the medication calculation test [5,6,7,8,9]. They question whether these levels are too low. One proposition to address the issue involves the requirement of a minimum level of mathematical skills for admission to nursing education [5,7]. However, a study conducted by Hutton [10] indicated that mathematical qualifications did not exert a significant effect on students’ ability to perform medication calculations.

Nurses are required to understand and apply key mathematical principles to administer the correct doses of medication [9]. Results of the study of Wright [9] indicated that nursing students lacked knowledge regarding the fundamental mathematical principles required in medication calculations. Brown [11] administered a basic numeracy test to more than 850 undergraduate nursing students across the United States of America (USA). The students were mathematically underprepared, particularly in skills involving fractions, decimals, and percentages. Lerwill [12] described attitudes to mathematics education in a group of healthcare students. The word most frequently chosen to describe feelings towards mathematics at school was “frustrating”. Lerwill [12] claimed that this indicated that mathematics education in schools was failing to meet students’ needs, regardless of whether they were interested in the subject. Gladhus and Grov [13] described similar findings. A high number of students used negative words to describe their relationships with mathematics; this included students with high expectations regarding mathematics mastery.

Røykenes and Larsen [7] investigated the relationship between students’ experiences of mathematics at school and their beliefs regarding their ability to pass the medication calculation test with a perfect score. The students who evaluated their mathematics knowledge as poor found the requirement to complete the test without error more stressful relative to students who judged their mathematics knowledge to be good. Newton, Harris, Pittilgo, and Moore [6] indicated that nursing students with low mathematical aptitude were less likely to pass the medication calculation test successfully on the first and subsequent attempts relative to students with higher mathematical aptitude. Findings from a study conducted in Finland indicated that students who evaluated their mathematical skills as sufficient were also successful with respect to dosage calculation [14]. Wright [9] claimed that students may have been more mathematically capable than they perceive themselves to be. Several studies have demonstrated that nursing students experience anxiety concerning mathematics [8,15,16]. Another study focused on students’ self-efficacy and mathematics examination grades [17]. Students with lower self-efficacy achieved lower marks relative to those with higher self-efficacy. Glaister [16] claimed that students’ anxiety should be taken into consideration in mathematics education, and arrangements should be tailored to individual students.

Grandell-Niemi, Hupli, Puukka, and Leino-Kipli [18] evaluated mathematical skills in 364 nurses and 282 graduating nursing students in Finland. The nurses and graduating nursing students completed the Medication Calculation Skills (MCS) Test. Nurses with an upper secondary school education exhibited superior performance in calculation problems relative to those with a lower basic education. The students lacked accurate mathematical skills, and those who had achieved excellent marks (9–10) in mathematics had studied mathematics for a longer period at high school and scored higher in the MCS Test relative to those who achieved lower marks. Another Finnish study found that high secondary school mathematics grades were associated with the ability to calculate correctly [14]. Kapborg [19] described similar findings, in that students with an upper secondary education achieved higher average scores relative to other groups.

A high proportion of students fail the medication calculation test on the first attempt; in Norway, this proportion is approximately 40% [5,20,21]. Students often require several attempts before passing the test. In a study conducted by Weeks, Lyne, and Torrance [21], nursing students were required to achieve a perfect score in the test. The results indicated that more than 50% of the students failed on the first attempt, and approximately 16%–33% failed on the second and third attempts. Blais and Bath [22] reported the results of a study conducted in the USA in which 89% of 66 first-year nursing students failed a 20-item drug calculation test with a 90% pass level. Jukes and Gilchrist [23] repeated these results in a UK study involving 37 second-year nursing students; in a 10-item drug calculation test, none of the students achieved full marks, 91% were unable to achieve the 90% pass mark, and 64% achieved marks of less than 70%. Gladhus and Grov [13] examined the relationships between students’ study assumptions (*i.e.*, expectations regarding mastering the test), levels of test anxiety, and test scores. The student group with high expectations of mastery did not achieve higher marks on the test relative to those with lower expectations and high levels of test anxiety.

Four types of error that nursing students commit in the test have been described in previous research [9,17,21,22,24]. The first type of error is conceptual, examples of which include lacking understanding of the logic of a problem and establishing the medication calculation equation incorrectly. The second type of error concerns arithmetical operation, examples of which include misunderstanding arithmetical operations such as division of a fraction denominator by the numerator. The third type of error involves computation, examples of which include failure to understand how to compute calculations and making basic multiplication and division errors. The fourth type of error concerns measurement such as that involved in the metric and apothecaries’ systems. Grandell-Niemi, Hupli, and Leino-Kipli [14] reported that students made mistakes with basic arithmetic and dosage calculations. Segatore, Edge, and Miller [25] examined whether preclinical baccalaureate nursing students could calculate at least 85% of medication doses correctly and subsequently described the nature and magnitude of their errors. The students failed to provide the correct medication or formulas. These errors occurred more frequently than mathematical errors such as incorrect addition, subtraction, multiplication, division, or use of decimals and fractions.

The aspects of the medication calculation test that second-year students felt more confident and less confident about have been described [17]. Students felt most confident in their ability to perform addition, subtraction, and division and least confident in their multiplication ability. They also lacked confidence in their ability to use fractions and ratios. They were most confident when asked to undertake simple mathematical calculations involving a minimal number of steps, such as those required to convert fluid volume from litres to millilitres. Less confidence was reported when students were asked to perform calculations involving multiple steps, such as those required to determine the number of tablets to be administered when the available medication stock consists of tablets of various strengths. In the UK, 229 second-year nursing students and 44 registered nurses attending a non-medical prescribing program completed validated numerical and drug calculation tests [26]. Both groups were significantly more able to perform calculations involving solids, oral liquid, and injections relative to those involving drug percentages and drip, or infusion, rates. Jukes and Gilchrist [23] explored nursing students’ ability to calculate mathematical drug problems at an English university; many were unable to perform drug calculations competently. Overall, students achieved the most correct answers in the section requiring an understanding of percentages. They demonstrated particular difficulty in the ratio and proportion section, in which they achieved the fewest correct answers. Wolf, Hicks, and Serembus [27] examined the characteristics of medication errors made by nursing students during the administration phase of the medication use process, which were reported to MEDMARX, a database operated by the United States Pharmacopeia via the patient safety program. Medication errors involving omission were reported most frequently, followed by those concerning administration of an incorrect amount of medication. The most prevalent cause of these errors was student performance deficits, while inexperience and distractions were leading contributory factors.

Wright [28] posited that there is little evidence demonstrating nurses’ poor medication calculation skills in practice. The problem lies in the way these skills are assessed. Several authors have discussed and suggested ways to meet the challenge of improving nursing students’ skills in medication administration and management [8,9,13,21,24,29,30,31,32]. Gladhus and Grov [13] concluded that a focus on teachers’ didactic repertoires is important in higher education, to increasing the likelihood that nurses will pass the medication calculation test. Teachers’ use of different approaches, such as textbooks, e-learning, teaching, math groups, close follow up, clinical case studies, and paying more attention to group participation have implications for students’ learning in the subject [13,29,30,31,32]. Wright [24] posited that there should be a focus on both mathematical and conceptual skills in learning. The main outcome of a review by Stolic [32] was that there are very few well-designed and adequately powered studies with a specific focus on learning strategies in undergraduate nursing education. Nevertheless, the conclusion reached from previous research is that access to different learning strategies helps students to master medication calculation.

Other studies focused on the importance of providing opportunities for nursing students to improve their mathematical skills while developing their knowledge regarding medication calculation in clinical practice [8,9,21,28,31]. Weeks, Lyne, and Torrance [21] concluded that students should understand both real-life medication problems, which are encountered in clinical nursing practice, and classroom-oriented abstract problems, formulae, and equations, which are used to represent, explain, and solve these problems. Walsh [8] claimed that students appeared to require time to mature in order to understand the gravity of performing accurate medication calculations in nursing practice and learn how to apply their mathematical skills. Wright [28] claimed that medication calculation should be examined and studied in clinical practice, where it is relevant and meaningful and immersed in the social practice of nursing. Nurse educators should consider the importance of problem representation in solving medication calculations, particularly with respect to ensuring that written questions are representative of clinical practice [33].

The literature review indicated that, in both Europe and the USA, ensuring that nurses and nursing students possess sufficient competence and skills to achieve a perfect score in the medication calculation test is a challenge. Previous research has focused on nurses’ and nursing students’ mathematical skills and medication management, the relationship between anxiety and passing the medication calculation test, and how to facilitate learning. Research has revealed that a belief in mathematics is a significant factor in passing the medication calculation test. Other studies have suggested that student mathematical skills and attitudes and challenges in the subject are significant factors; however, these studies did not provide explicit information. Research concerning the significance of mathematics in performing correct calculations is not sufficiently consistent or clear. Some studies imply that mathematical qualifications could be associated with the ability to calculate correctly, while others describe contradictory findings. There is a lack of research concerning the relationship between mathematical qualifications and the number of attempts required to pass the medication calculation test. Knowledge regarding this relationship is of importance in meeting the challenges nursing students face in learning medication calculation and management. Therefore, the purpose of this pilot study was to explore the relationship between high school mathematics grade and the number of attempts required to pass the medication calculation test in nurse education.

## 2. Methods

### 2.1. Participants

The study used an exploratory design. Using questionnaires and statistical analysis, we sought to explore the relationship between high school mathematics grade and the number of attempts required to pass the medication calculation test. The inclusion criterion was that the students had passed the test. One hundred and ten nursing students from three classes in the Bachelor of Nursing program at a university college in Norway were invited to participate in the study. The participant group consisted of men and women aged 18–50 years.

### 2.2. Teaching in Medication Calculation

As preparation, the nursing students attended lectures concerning the topics included in the test. They calculated and solved mathematical problems in supervised groups. The supervision was intended to address individual students’ problems and challenges concerning medication calculation. In addition to the lectures and mathematical problem solving, the students measured tablet dosages using a pill organizer in the clinical laboratory at the university college.

In the medication calculation test, students solved mathematical problems concerning medication management. They performed calculations according to measurement units, dosage calculation, strength, amount, infusion volume, and infusion rate. They performed further calculations using time, decimals, percentages, and liquid pharmaceutical dilution. To perform the medication calculations, students required mathematical skills in addition, subtraction, multiplication, division, basic functions with decimals, fraction computation, percentages, calculate an equation with an unknown, and knowledge of ratios and proportions. In Norway, students acquire the required mathematical knowledge in elementary school.

The exam was a 20-item medication calculation test. In the exam, students demonstrate that they have mastered error-free medication calculation. Each time students complete the exam, their knowledge of the subjects described is tested. The students perform different tasks in different exams.

### 2.3. Students’ Questionnaire Access

Three class teachers at the university college informed the students about the study and distributed the questionnaire. The information was based on an information sheet developed in advance by the researchers. The class teacher informed the students about the study purpose and procedure, and discussed the relevant ethical issues. The students who wished to participate completed the questionnaire and submitted it to their class teacher.

### 2.4. The Questionnaire

We developed a questionnaire to determine the students’ high school mathematics grades and the number of attempts they had required to pass the medication calculation test. The questionnaire was developed based on findings from previous research and our experiences in teaching medication calculation and management over several years. We considered the questionnaire understandable and clear.

The questionnaire consisted of four questions. In the first question, students were asked if they had attained mathematics qualifications at a level equivalent to that of high school. If they answered “yes” to this question, they were asked to indicate the highest mathematics grade (1–6) they had completed in high school. Grades 1 and 6 were the lowest and highest levels, respectively, and grade 2 was the lowest passing grade.

If the nursing students answered “no” to this question, indicating that they had not attained mathematics qualifications at a level equivalent to that of high school, they were asked to indicate which grade they had completed in secondary school (aged 13–16 years). As all of the students indicated that they had high school mathematics qualifications, the question concerning secondary school grades was irrelevant. Finally, they were asked to provide details of the number of attempts they had required to pass the medication calculation test at the university college.

In retrospect, the questionnaire was not sufficiently detailed with respect to the descriptions of the participating students, their mathematical skills, the time required to acquire their skills, and the grade specified in the questionnaire. The students had completed at least two mathematics grades in high school, one from the first year and the other from the second year. In addition, they could have completed an examination grade and the third-year grade in high school.

### 2.5. Ethics

The questionnaire included no personal identifying information. The students consented to participation by completing the questionnaire. We did not produce a list of participants, their responses were anonymous, they completed the questionnaire voluntarily, and we did not send reminders. Therefore, the study was not notifiable. However, because it was a part of a larger study, it was reported to the Norwegian Social Science Data Services.

### 2.6. Statistics

We developed a data matrix to simplify and summarize the number of attempts required to pass the test. We distributed the results for the two variables: High school mathematics grade and the number of attempts required to pass the medication calculation test. Subsequent to recording the numbers of students who had completed grades 2, 3, 4, and 5 in high school mathematics, we counted the number of times each result was repeated. The results for each variable in the data matrix were then compared. These results were presented as numbers and percentages (Table 1).

To explore the correlation between high school mathematics grade and the number of attempts required to pass the medication calculation test, we also performed chi-squared and Fischer’s exact tests [34,35]. Grades 2 and 3 (lowest) and grades 4 and 5 (highest) were compared according to whether the students had passed the test on the first attempt or the second, third, or fourth attempt (Table 2). The chi-square test results were calculated manually. The significance level was set at *p* < 0.05.

## 3. Results

Of the 110 questionnaires distributed, 90 were returned (response rate of 82%). All of the students (*n* = 90) indicated that their mathematical skills were equivalent to high school level. None of the students had completed high school grades 1 or 6; 16 (18%), 36 (40%), 28 (31%), and 10 (11%) students had completed grades 2, 3, 4, and 5, respectively. Of the 90 participants, most (40%) had completed grade 3, and 52 (58%) had completed grade 3 or lower.

On the first attempt, regardless of the mathematics grade completed, 43% (*n* = 39) of the students passed the medication calculation test. By the second attempt, 82% (35 additional students) had passed, by the third attempt, 89% (six additional students) had passed, and by the fourth attempt, 93% (four additional students) had passed (Table 1).

Six (7%) of the students failed the test on their fourth attempt. Of these, students 1, 3, and 2 had completed grades 2, 3, and 4, respectively. This illustrates that the students who failed on their fourth attempt had completed various grades, excluding grade 5, as all of the students who had completed grade 5 had passed the test on the third attempt.

**Table 1:** The relationship between high school mathematics grade and the number of attempts required to pass the medication calculation test.

High School Mathematics Grade	Passed on 1st Attempt *n* (%)	Passed on 2nd Attempt *n* (%)	Passed on 3rd Attempt *n* (%)	Passed on 4th Attempt *n* (%)	Failed on 4th Attempt *n* (%)	Total *n* (%)
2	7 (44)	6 (38)	1 (6)	1 (6)	1 (6)	16 (100)
3	15 (42)	13 (36)	2 (6)	3 (8)	3 (8)	36 (100)
4	11 (39)	14 (50)	1 (4)	0	2 (7)	28 (100)
5	6 (60)	2 (20)	2 (20)	0	0	10 (100)
Total	39 (43)	35 (39)	6 (7)	4 (4)	6 (7)	90 (100)

**Table 2:** Numbers of students who passed on the first and second, third, and fourth attempts, used in the chi-square and Fisher’s exact tests.

High School Mathematics Grade	Passed on 1st Attempt *n*	Passed on 2nd, 3rd, or 4th Attempt *n*	Total *n*
2–3	22	30	52
4–5	17	21	38
Total	39	51	90

The chi-square test p value was 0.052. This was slightly higher than the level of significance (*p* < 0.05), suggesting that a correlation between high school mathematics grade and the number of attempts required to pass the medication calculation test would be unlikely.

The number of observations in the study was 90, which could be considered relatively low. As Table 1 demonstrates, there were fewer than five observations in some of the table squares. The chi-square test may therefore have provided a false conclusion. Fisher’s exact test is suitable when there are few observations and we have a 2 × 2 table [34].

Fisher’s exact test was performed to calculate an exact *p* value. The two-sided p value in Fisher’s exact test was 0.832, which confirms and strengthens the result of the chi-square test. Therefore, there was no statistically significant correlation between grade and the number of attempts required.

## 4. Discussion

The statistical calculations provided no basis for concluding that there was a statistically significant correlation between high school mathematics grade and the number of attempts required to pass the medication calculation test. The figures in Table 1 also illustrate this. Table 1 shows that students who had completed grades 2, 3, 4, and 5 passed the medication calculation test on the first attempt. The students’ mathematics grades were a measure of their ability and skills in the subject. A student who has completed grade 5 is expected to possess stronger mathematical abilities relative to students who have only completed grade 2. Based on their grades, there is therefore an expectation that students who have completed high grades have a greater opportunity to pass the test on the first attempt relative to students who have completed lower grades. This expectation was confirmed in a study conducted by Newton, Harris, Pittilgo, and More [6]. Students with higher mathematics ability scores were more likely to pass the medication calculation test on the first attempt relative to students whose mathematical abilities were weaker. Three other studies [14,18,19] reported similar findings. Students with higher levels of mathematical education tended to achieve higher scores in the medication calculation test. In this study, a larger proportion (60%) of the students who had completed grade 5 had passed the medication calculation test on the first attempt relative to those who had completed lower grades; 44%, 42%, and 39% of students who completed grades 2, 3, and 4, respectively, passed on the first attempt.

Which mathematical knowledge is required to solve the mathematical problems in the test? The students who participated in this study acquired the required mathematical knowledge while in elementary school. Therefore, a high level of mathematical education should not be a significant factor. Why then, do students who have completed higher grades tend to score higher in the medication calculation test? One explanation may be that students with higher levels of mathematical education have repeated the required knowledge at school over a higher number of years relative to those with lower levels of mathematical education. Repetition is a learning strategy whereby students develop and maintain their knowledge.

Eighty-two percent of the students, regardless of the mathematics grade completed, had passed on the second attempt. All of the students who had completed grade 5 had passed by the third attempt, and all of the students who had not passed after four attempts had completed grades lower than grade 5. This may indicate that students who had not completed grade 5 required a higher number of attempts to pass relative to those who had achieved a higher level of education. Nevertheless, there were students who had completed grade 5 who required three attempts, while some students who had completed grade 2 passed the medication calculation test on the first attempt. The students who failed on the fourth attempt had completed grades 2, 3, or 4. These results demonstrate that grade is not a crucial factor in passing the test and reduces the importance of mathematics at a higher level than that of elementary school.

Fifty-seven percent of the students failed to achieve a perfect score on the test on the first attempt. This proportion is higher relative to the proportions of 40% [5,20] and more than 50% [21] reported in other studies. However, it is lower relative to the proportions reported in studies conducted by Blais and Bath [22] and Jukes and Gilchrist [23], in which approximately 90% of the students were unable to achieve a mark of 90%. With a high rate of failure on the first attempt, students require additional attempts to achieve perfect scores in the medication calculation test. Allowing for several attempts entails additional resource consumption for students and universities or university colleges. Previous research has recommended that learning should include mathematical concepts as well as application of mathematical skills in clinics, where nursing students face problems and challenges related to handling medication [8,9,21,28,33]. The students who participated in this study were required to pass the test before they could handle drugs in the clinic. Learning calculation in clinical environments was not part of the test preparation activity. The goal was to pass the medication calculation test within fewer attempts.

The medication calculation test is not a mathematics exam. It is designed to allow students to demonstrate that they have mastered error-free medication calculation. Mathematics received an overriding focus, as it has in previous research [5,6,7,8,9,10,11,12,13,14,15,16,17,18,19,21,22,24,25]. Which knowledge do educational institutions wish to secure via the examination? Do the students achieve the skills necessary for handling medicines in the clinic? In research, the errors that nursing students commit in the test are described as mathematical [9,14,17,21,22,24,25]. It is lack of research concerning the understanding of central concepts within medication management such as those surrounding the amount, dosage and strength, and form of medication. An interesting question in this discussion is that of the extent to which understanding the meaning of these concepts is important in passing the medication calculation test. This is an area that requires further exploration.

## 5. Limitations of the Study

This study involved nursing students from three different classes at a university college in Norway. Ninety students participated in the study, which is a small number relative to the wider population of students in Norway. The small numbers of students shown in each table square may have exerted a greater impact on the proportional distribution of data, creating a stronger bias with respect to the proportions reported. For example, the students who had completed grade 5 represented 11% of the 90 students. A larger number of students may have produced a statistical difference. This also applies to the types of mathematics specialization in high schools. However, as the numbers in the table and the results of the statistical tests demonstrate, there was little or no difference between the grades.

One weakness of this study was the development of the questionnaire. We did not specifically ask students to indicate the choices they had made with respect to the mathematics studied in high school. Therefore, we did not collect information regarding students’ mathematical skills. In the questionnaire, all of the students indicated that they had attained mathematics qualifications equivalent to that of high school. For students who attended high school prior to 1994, the study of mathematics was obligatory during the first year; in the second and third years, students could choose mathematics specialization. Students who attended high school subsequent to 1994 studied mathematics for five hours per week in the first year, and they could choose between ‘practical mathematics’ (Vg1P) and ‘theoretical mathematics’ (Vg1T). In the second year of high school, students were required to study mathematics for a minimum of three hours per week. We do not know whether the students who chose Vg1T also chose a mathematics specialization in the second and third years. Mathematics specialization involved study for five hours per week. The small number of students and lack of knowledge regarding their mathematical choices in high school was perceived as a random variation. In addition, the questionnaire was not scientifically validated, which had implications for the validity of the study.

The students completed the questionnaires themselves. The participating students were aged 18–50 years, and many had left high school more than four years prior to the study. They may have forgotten or misremembered details concerning the highest grade completed. Some may have provided incorrect information regarding grades or reported higher or lower grades than those actually completed. The collection of more specific information could have provided knowledge regarding whether students who chose a specialization fared better on the test relative to those who did not.

One of the strengths of the study was the high response rate of 82% and the low proportion of non-responders. With respect to the 20 students (18%) who chose not to participate, we had no information regarding their grades or the number of attempts they required to pass the test. Therefore, we cannot comment regarding how their responses would have affected results. In addition, there have been no similar studies conducted with which to compare the results.

## 6. Conclusions and Future Directions

High school mathematics grade and the number of attempts required to pass the medication calculation test in nurse education were not significantly correlated. Mathematics grades may have been important in ensuring a pass within fewer attempts; however, their importance was not crucial. Students with both high and low grades had passed the test on the first attempt. The relationship between the high school mathematics grade and the number of attempts required to pass the medication calculation test is complicated and challenging. The high school mathematics grade was not critically important, as there were a number of extraneous influencing factors.

The correct use of medication at the right time is important in ensuring patient health. Many resources, both financial and personal, are used to find appropriate means of approaching challenges in students’ learning situations. This pilot study provided some answers. Because the study was conducted in one university college in Norway, the results were not necessarily representative of the wider population. In order to provide appropriate answers regarding the relationship between high school mathematics and the number of attempts required to pass the medication calculation test, it is necessary to replicate the study. In a replication study, the student population should be expanded to include educational institutions both inside and outside Norway. Moreover, the questionnaire should be expanded to provide more nuanced answers, particularly with respect to differences in students’ levels of mathematical skills and whether their mathematical skills are important in passing the medication calculation test. Another perspective reported in previous research is that of the importance of understanding the key concepts of medication management in order to pass the test. Further research expanding this subject is required.
